# The Role of Cognition, Personality, and Trust in Fraud Victimization in Older Adults

**DOI:** 10.3389/fpsyg.2017.00588

**Published:** 2017-04-13

**Authors:** Rebecca A. Judges, Sara N. Gallant, Lixia Yang, Kang Lee

**Affiliations:** ^1^Department of Applied Psychology and Human Development, University of Toronto, TorontoON, Canada; ^2^Department of Psychology, Ryerson University, TorontoON, Canada

**Keywords:** scams, cognitive ability, fraud, victimization, aging, older adults

## Abstract

Older adults are more at risk to become a victim of consumer fraud than any other type of crime (Carcach et al., 2001) but the research on the psychological profiles of senior fraud victims is lacking. To bridge this significant gap, we surveyed 151 (120 female, 111 Caucasian) community-dwelling older adults in Southern Ontario between 60 and 90 years of age about their experiences with fraud. Participants had not been diagnosed with cognitive impairment or a neurological disorder by their doctor and looked after their own finances. We assessed their self-reported cognitive abilities using the MASQ, personality on the 60-item HEXACO Personality Inventory, and trust tendencies using a scale from the World Values Survey. There were no demographic differences between victims and non-victims. We found that victims exhibit lower levels of cognitive ability, lower honesty-humility, and lower conscientiousness than non-victims. Victims and non-victims did not differ in reported levels of interpersonal trust. Subsequent regression analyses showed that cognition is an important component in victimization over and above other social factors. The present findings suggest that fraud prevention programs should focus on improving adults’ overall cognitive functioning. Further investigation is needed to understand how age-related cognitive changes affect vulnerability to fraud and which cognitive processes are most important for preventing fraud victimization.

## Introduction

The world’s population is graying. The World Bank reports that in 2014, 16% of the population in high income countries was 65 years of age or older (World Bank, 2015). While most research focuses on seniors’ health status and behaviors, all areas of older adults’ lives deserve attention. One such area is the issue of being victimized by consumer fraud, a type of crime that is most likely to be committed against older adults (Carcach et al., 2001). It may be particularly damaging for an older person to be a victim as they tend to be on fixed incomes (Ross et al., 2014). There is also the risk of losing their independence if others notice they are not making safe financial decisions (Ross et al., 2014). Many studies have examined the various demographic profiles that may make someone vulnerable to being scammed, with mixed and inconclusive findings (Titus et al., 1995; Moore and Lee, 2000; Anderson, 2004, 2013; Pak and Shadel, 2011; Deevy et al., 2012). For example, Titus et al. (1995) did not find any significant race effects when it came to fraud victimization, but other studies from the Federal Trade Commission (FTC) have shown that Aboriginal Americans, African Americans, and Hispanic Americans are more likely than Non-Hispanic White Americans to fall victim to fraud (Anderson, 2004, 2013). In contrast, only a handful of studies have examined psychological factors contributing to fraud victimization, such as major negative life events and risk-taking, again with mixed findings (e.g., Van Wyk and Benson, 1997; Consumer Fraud Research Group, 2006; Schoepfer and Piquero, 2009; Pak and Shadel, 2011; Anderson, 2013; Shadel et al., 2014). The current study aimed to bridge this significant gap in the literature by focusing on the role of cognitive, personality, and trust variables in relation to fraud victimization.

Cognitive theories of aging point to declines in biologically based cognitive functioning, such as the general slowing hypothesis (Salthouse, 1996) and the inhibitory deficit hypothesis (Hasher and Zacks, 1988). Indeed, research has consistently shown systematic declines in such areas as memory, processing speed, problem solving, mathematical skills, language, and executive functioning (Cohen, 1979; Nyberg and Bäckman, 2001; Verhaeghen and Cerella, 2002; Murphy et al., 2006; Thornton and Light, 2006; Kvavilashvili et al., 2009; Ross et al., 2014). These cognitive declines have been linked to poor financial decision making and financial literacy skills (Agarwal and Mazumder, 2013; Gamble et al., 2014; Wilson et al., 2016). For example, better overall cognitive performance and specifically better math skills are related to fewer mistakes when making household financial decisions (Agarwal and Mazumder, 2013). Gamble et al. (2014) also showed that declines in cognition over a period of 3 years were associated with declines in financial literacy (i.e., a combination of numeracy skills and financial knowledge). But as Wilson et al. (2016) state, the direction of the relationship between literacy and cognitive health can still be debated. Fluid intelligence declines have also been linked to worse financial decision making performance, although crystallized intelligence can make up for these deficits depending on the task (Li et al., 2013). These existing findings suggest that cognitive factors may play an important role in vulnerability to fraud victimization, but the research connecting specific cognitive and neural factors to fraud vulnerability has been scarce. One such study that has directly measured this is Asp et al. (2012). Following previous research that suggested that individuals with damage to the ventromedial prefrontal cortex (vmPFC) tend to be gullible and suggestible, the authors examined their own theory that the vmPFC is involved in “tagging” questionable ideas as doubtful. As the authors expected, those with damage to the vmPFC were more credulous to misleading advertisements compared to normal controls and those with brain damage in other areas. The subjects in Asp et al.’s study were adults of all ages, however, there is other work linking age-related changes in risky decision making with the vmPFC in cognitively intact older adults (Rogalsky et al., 2012). Taken together, these findings suggest that neural and cognitive factors will play an important role in fraud vulnerability in healthy older adults.

In terms of personality variables, agreeableness is characterized by a tendency to cooperate with others (Ashton and Lee, 2009) and may be linked to susceptibility to being manipulated (Pinsker et al., 2009). Though there is no empirical evidence of this relationship, those who are more willing to co-operate may be more likely to co-operate with those who are attempting to defraud them. Conscientiousness includes being detail-oriented and careful to consider the consequences of one’s actions (Ashton and Lee, 2009). A conscientious person may wish to take time to consider their financial actions and thus be less likely to be victimized. Honest individuals may be at risk of fraud because evidence shows that adults and children who behave honestly tend to expect others to behave honestly (Rotter, 1980; Evans and Lee, 2014). Alternatively, it is possible that honest individuals have a heightened sensitivity to fairness (Ashton and Lee, 2008), which could protect them against victimization because fraud schemes often involve unfair practices.

When considering the role of trust, only one study has considered trust in relation to fraud, which found that trust was significantly lower in lottery victims than it was in the general population (Anderson, 2003). This is surprising as common sense would suggest that those who are more trusting would be more at risk for fraud. Alternatively, recent trust research suggests those who are more trusting are better at differentiating between individuals who are trustworthy or untrustworthy (Carter and Weber, 2010).

The present study aimed to bridge this significant gap in the literature. Taken together, there is evidence to suggest that cognitive ability, personality, and degree of trust should play a significant role in the likelihood of experiencing fraud during aging; however, there has yet to be a study to specifically address this question. To this end, we examined the linkage between cognitive, personality, and trust factors and fraud victimization in an older adult population. Based on the existing cognitive aging research, we hypothesized that relative to non-victims, victims would perform worse on language, verbal memory, and attention measures, and subsequently overall cognitive ability. With regard to personality, it is expected that victims would be more agreeable (Pinsker et al., 2009) but less conscientious than non-victims (Lee and Ashton, 2004). Regarding honesty-humility, there are two possibilities: honest individuals may be more likely to be victimized due to their optimistic expectation of others’ honesty (Evans and Lee, 2014) or they may be protected by their desire to engage in fair behaviors (Ashton and Lee, 2008). Finally, with regard to trust, there are also two possibilities: those who are highly trusting may be more likely to be victims of fraud due to their tendency to trust others, but alternatively they may be better able to discern whether a potential fraudster is trustworthy or not (Carter and Weber, 2010).

## Materials and Methods

### Participants

The participants in this study were 174 older adults recruited from local communities in Southern Ontario. Participants were recruited in one of two ways: through flyers or short presentations at local community centers, or through The Ryerson Senior Participant Pool (RSPP). We recruited participants who had no diagnosis of a neurological disorder including any diagnosis of cognitive impairment by their doctor. Additionally, participants had to be 60 years of age or older, and be in charge of their own finances. Despite advertising this and including these criteria on our consent form, 12 participants were excluded at the analysis level due to not meeting study requirements. An additional six were excluded for large amounts of missing data and five were excluded because their reports of fraud victimization occurred before the age of 50. This was done to ensure that the fraud occurred when the person was older, but provided a reasonable window of 10 or more years for younger participants to have been a victim of fraud. The final analysis was completed with 151 participants (*M* = 74.33 years, *SD* = 6.19, age range = 61.6 to 88.9 years). The demographic make-up of the entire sample and the sample divided into victims and non-victims is displayed in **Table 1**.

**Table 1:** Participant characteristics as percentages of the sample.

Characteristic	Total (*N* = 151)	Victims (*n* = 51)	Non-victims (*n* = 100)
Gender			
Female	79.5 (*n* = 120)	78.4 (*n* = 40)	80.0 (*n* = 80)
Male	20.5 (*n* = 31)	21.6 (*n* = 11)	20.0 (*n* = 20)
Education			
High school or less	22.5	21.6	23.0
Bachelor or equivalent	32.5	33.3	32.0
Post-graduate education	13.9	15.7	13.0
Missing responses	31.1	29.4	32.0
Years in Canada			
Under 50 years	34.7	42.0	30.9
50 years and more	62.7	56.0	66.1
Missing responses	2.6	2.0	3.0
First Language			
English	63.6	62.7	64.0
Other	21.9	21.6	22.0
Missing responses	14.6	15.7	14.0
Ethnicity			
Aboriginal	0.7	2.0	-
Black	4.6	5.9	4.0
East Asian	6.0	11.8	3.0
Latin American	2.0	3.9	1.0
South Asian	4.6	2.0	6.0
South East Asian	4.6	3.9	5.0
Caucasian	73.5	66.7	77.0
Other (mixed)	3.3	2.0	4.0
Missing responses	0.7	2.0	-
Living arrangement			
Alone	51.0	49.0	52.0
With family	23.2	25.5	22.0
With significant other	21.9	23.5	21.0
In a retirement residence	3.3	2.0	4.0
Other	0.7	-	1.0
Living environment			
Small town	4.0	3.9	4.0
Mid-large town	2.6	-	4.0
Small city	15.9	13.7	17.0
Mid-large city	72.8	80.4	69.0
Missing responses	4.6	2.0	6.0


*Forty-seven missing responses from education variable due to procedural error.*

### Measures

All participants completed the same five measures in the following order:

#### Demographics

This was an 11-item measure which asked about age, gender, education, location (to examine rural, urban, suburban living), how long they have lived in Canada for, living arrangement, English as a first language (or other languages they speak), and average time they spend in a week with various groups (divisions based on closeness and age).

#### Fraud Questionnaire

Following this, they completed a fraud questionnaire developed for this study. First, participants are asked if they have ever been a victim of fraud, allowing participants to self-identify with their own fraud definition. Then participants are asked about 15 different types of fraud, chosen to provide a sample of the most common fraud schemes and those that are specifically targeted to older adults (National Fraud and Cyber Crime Reporting Centre, 2011; Anderson, 2013; Government of Canada, 2013). The 15 scams were as follows: weight loss scam, miracle health product scam, prize or lottery fraud, fraudulent work-at-home programs, charity scam, credit repair fraud, fraudulent business opportunity, advance free loan scam, counterfeit check scam, clairvoyant scheme, phishing, inheritance fraud, timeshare scam, emergency or grandparent scam, and fraud where items purchased are never received or are incorrect. All fraud scenarios include questions about whether they have ever been approached by this type of fraud, whether they had been victimized, the method of contact (i.e., internet/e-mail, telephone, in-person, other), the age they were when the fraud occurred, and the amount of money that they lost (see Appendix).

#### Multiple Ability Self-report Questionnaire (MASQ)

The MASQ is a self-report measure which looks at cognitive ability (Seidenberg et al., 1994). The full questionnaire (Cronbach’s alpha = 0.90) includes five scales: language, visual-perceptual ability, verbal memory, visual-spatial memory, and attention/concentration (Seidenberg et al., 1994). For our purposes, only responses on the language, verbal memory, and attention/concentration scales were collected, and had Cronbach’s alphas of 0.75, 0.79, and 0.73, respectively. The measure included statements such as “I find myself searching for the right word to express my thoughts.” Participants were asked to rate how well these statements described them on a scale from 1 to 5 with higher scores indicating greater cognitive ability.

#### Trust Survey

Next participants completed our trust survey, which used seven items measuring trust from the World Values Survey (World Values Survey Association, 2012). This includes a general trust question where participants were asked if they generally believed that others were trustworthy or untrustworthy. They were also asked to rate their level of trust from 1 (trust completely) to 5 (do not trust at all) for six different groups including family, friends, neighbors, strangers, and people of different religions and nationalities. The Cronbach’s alpha for these six questions was 0.81, and they were added together to create a total trust score.

#### The HEXACO Personality Inventory

The final questionnaire was the 60-item HEXACO-PI (Ashton and Lee, 2009). It included six subscales with the following Cronbach’s alpha for each: honesty-humility (0.53), emotionality (0.67), extraversion (0.70), agreeableness (0.65), conscientiousness (0.68), and openness to experience (0.75). This specific personality scale was chosen over other personality measures due to its inclusion of the honesty-humility trait. The inventory includes statements like “In social situations, I’m usually the one who makes the first move” (extraversion, social boldness subscale). Each domain included 10 statements, and participants rated how true or untrue these statements were of them on a scale from 1 to 5.

### Procedure

The study was approved by the Research Ethics Boards at the University of Toronto and Ryerson University. All participants completed informed consent prior to participating. Participant data was anonymized using an assigned participant number which was used throughout analysis. Approximately two thirds of the participants completed the questionnaires at home, on their own time, and then mailed the completed package back to our lab. The remaining third of the participants completed the study in a group setting with the researchers present. The organizers at the community centers advertised the study date through flyers and managed the sign-up of interested older adults. At each session, 2–3 researchers were present to field questions and make sure the session ran smoothly.

Those who agreed to participate were mailed a questionnaire package. Interested community centers could also schedule a date and time with our lab to come in and conduct the survey. Out of the final 151 participants, 111 participants were mailed the study and 40 completed the study at a local community center. There were no significant differences between these groups on any of the measures.

## Results

### Univariate Analysis of Demographic Characteristics of Victims and Non-victims

Out of the sample of 151 participants, 51 individuals (34%, *M*_age_ = 73.47, *SD* = 5.25) indicated that they had been victimized by fraud. The demographic characteristics of the victim and non-victim groups are displayed in **Table 1**. Independent *t*-tests were conducted for each demographic variable to compare victims and non-victims (also see **Table 1**). All statistical analyses utilized an alpha level of 0.05 and were conducted using IBM SPSS version 22. Although there were some slight differences between the groups (e.g., there were more Caucasian participants and participants who lived in larger cities in the non-victim group), these differences were not significant (*p*s > 0.05).

### Univariate Analysis of the Psychological Characteristics of Victims and Non-victims

**FIGURE 1:**
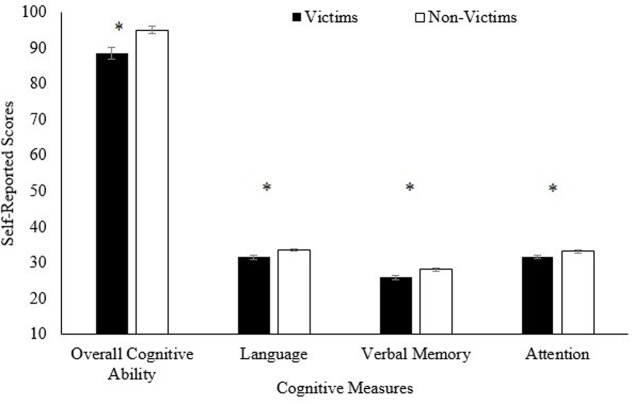
**Mean self-reported scores on the MASQ (cognitive ability questionnaire), comparing victims and non-victims.** The overall cognitive ability score ranged from a possible 24 to 120 points. The three subscales ranged from a possible 8 to 40 points. Standard errors are represented by the error bars attached to each column. ^∗^*p* < 0.05.

**FIGURE 2:**
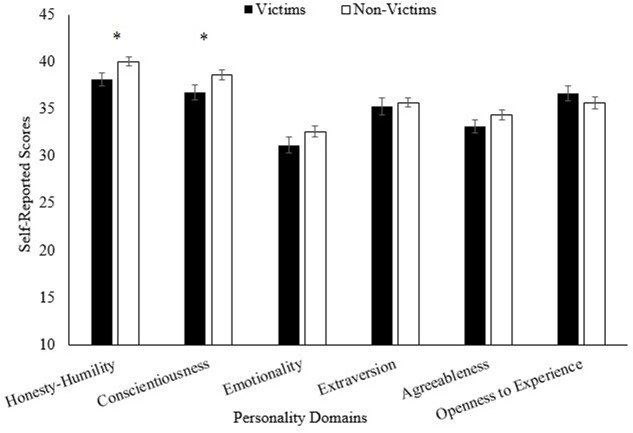
**Mean self-reported scores on the HEXACO-PI (personality questionnaire), comparing victims and non-victims.** All scores ranged from a possible 10 to 50 points. Standard errors are represented by the error bars attached to each column. ^∗^*p* < 0.05.

Independent sample *t*-tests were used to determine differences between victims and non-victims on the psychological variables. This parametric test was used as all the psychological variables were normally distributed according to the Shapiro–Wilk test (*p*s > 0.05) with the exception of language (*p* = 0.017) and verbal memory (*p* = 0.049) for the non-victim group. The differences between the two groups in cognitive abilities and personality traits are shown in **Figures 1**, **2**, respectively. Overall cognitive ability (language, verbal memory, and attention scores combined) was significantly higher in non-victims than in victims, supporting our hypothesis. This significant difference was present at all levels of the cognitive measures, with language, [*t*(145) = 3.03, *p* = 0.003, *d* = 0.52], verbal memory, [*t*(135) = 3.33, *p* = 0.001, *d* = 0.63], and attention scores, [*t*(136) = 2.21, *p* = 0.029, *d* = 0.40] all significantly higher in the non-victim group compared to the victim group (see **Figure 1**). Overall cognitive ability was also significantly higher in non-victim group, *t*(128) = 3.45, *p* = 0.001, *d* = 0.65.

Among all the personality variables (see **Figure 2**), two domains had significant differences: honesty-humility, [*t*(136) = 2.15, *p* = 0.033, *d* = 0.39], and conscientiousness, [*t*(140) = 2.09, *p* = 0.038, *d* = 0.036]. Victims were significantly less honest and humble than non-victims supporting the hypothesis that honest individuals would be less likely to get involved with fraud schemes. The victims were also significantly less conscientious than non-victims, supporting our hypothesis regarding conscientiousness. There were no significant differences in agreeableness between the two groups, *t*(140) = 1.37, *p* = 0.173, *d* = 0.24.

For generalized trust, there were two measures. For the generalized trust item, the victims (*M* = 1.26, *SD* = 0.44) and non-victims (*M* = 1.38, *SD* = 0.49) were not significantly different, *t*(108.91) = 1.53, *p* = 0.129, *d* = 0.26. The Levene’s test was significant (*F* = 10.41, *p* = 0.002) so equal variances was not assumed. Using a composite trust score of trust across the six groups of people, victims had a mean score of 14.10 (*SD* = 3.12) and non-victims had a mean score of 13.63 (*SD* = 2.92) out of a possible 25, but this was also not significantly different, *t*(142) = 0.50, *p* = 0.37, *d* = 0.16.

### Logistic Regression Analysis of Psychological Characteristics of Victims and Non-victims

To examine the extent to which the above significant factors contribute to being victimized, we performed two logistic regression analyses. Because a preliminary logistic analysis with the age and gender control variables as predictors and fraud victimization as the predicted variable yielded no significant effects, these two control variables were not further considered in the following formal logistic regression analyses.

The first regression analysis included the personality traits honesty-humility and conscientiousness, as well overall cognitive ability as predictors to see impact of personality and cognitive differences in predicting fraud victimization. The model was significant and the variables together accounted for 18% of the variance in victimization, Nagelkerke Δ*R*^2^ = 0.18, χ^2^ (3, *N* = 121) = 16.56, *p* = 0.001. According to the general rule of thumb for VIF, these values are acceptable and do not cause concern for multicollinearity (O’Brien, 2007).

Further inspection of the model (see **Table 2**) revealed that honesty-humility and conscientiousness were not significant and that each did not uniquely predict fraud victimization above and beyond the common contributions of all the three predictor variables. However, overall cognitive ability was significant, suggesting that it uniquely predicted fraud victimization above and beyond the common contributions of all the predictors. The mean odds ratios showed that for every 10-point decrease in the overall cognitive ability score, one is approximately 11 times more likely to be a victim of fraud.

**Table 2:** Factors predicting fraud victimization using overall cognitive ability.

Predictors	*B*	*SE*	Wald	OR [95% CI]	VIF
Cognitive ability	0.08	0.03	8.42^∗∗^	0.93 [0.88–0.98]	1.39
Honesty-humility	0.01	0.05	0.09	0.99 [0.90–1.08]	1.11
Conscientiousness	0.03	0.05	0.28	0.98 [0.89–1.07]	1.33


*^∗∗^*p* < 0.01.*

Given the significant findings of the above regression analysis, in particular the significant unique contribution of overall cognitive ability, we further explored whether any of the specific cognitive skills that made up the overall cognitive ability score were predictive of fraud victimization. To do so, we ran a second logistic regression with the two personality traits, as well as the three cognitive subscales of language, verbal memory, and attention as predictors. The model was significant, Nagelkerke Δ*R*^2^ = 0.19, χ^2^ (5, *N* = 121) = 17.17, *p* = 0.004 and altogether the variables accounted for 19% of the variance in fraud victimization. As shown in **Table 3**, according to the general rule of thumb for VIF, these values are acceptable and do not cause concern for multicollinearity (O’Brien, 2007). However, inspection of the model revealed that none of these individual cognitive variables uniquely predicted fraud victimization above and beyond the common contributions of all predictor variables (*p*s > 0.10, see **Table 3**).

**Table 3:** Factors predicting fraud victimization using cognitive subscales.

Predictors	*B*	*SE*	Wald	OR [95% CI]	VIF
Language	0.10	0.07	2.00	0.90 [0.78–1.04]	1.96
Verbal memory	0.12	0.09	1.87	0.89 [0.75–1.05]	2.34
Attention	0.00	0.10	0.00	1.00 [0.82–1.21]	2.86
Honesty-humility	0.02	0.05	0.21	0.98 [0.90–1.07]	1.15
Conscientiousness	0.03	0.05	0.28	0.98 [0.89–1.07]	1.36


## Discussion

The present study examined the role of cognitive, personality, and trust factors in fraud victimization among older adults. The univariate analyses suggest that cognitive ability, conscientiousness, and honesty-humility were lower in victims than non-victims. This was consistent with our hypotheses regarding the role of cognitive factors and conscientiousness in fraud victimization whereas the honesty-humility findings support the hypothesis that honesty may be a potential protective factor. Other personality traits and trust toward others did not differ significantly between the victimization groups. With the logistic regression, overall cognitive ability, honesty-humility, and conscientiousness collectively were significantly associated with whether an individual was a victim or not. Furthermore, overall cognitive ability predicted fraud victimization above and beyond the personality variables. When the overall cognitive ability was broken down into the three specific cognitive subscale scores, none of them were uniquely associated with fraud victimization above and beyond the common contributions of all predictor variables. Below we discuss these major findings.

### Group Differences

#### Cognitive Factors

The univariate *t*-tests showed that overall cognitive ability was lower in victims than non-victims, and the same pattern was displayed for the language, verbal memory, and attention/concentration subscales. Cognitive functioning declines may increase the likelihood of being targeted by those who notice signs of confusion (MetLife, 2011). Declines in global cognitive ability have been linked to decreases in financial literacy, which is key to making sound financial decisions (Wilson et al., 2016). Previous research has shown that normal age-related changes to the vmPFC is linked to poor decision making in healthy adults and individuals with damage to this brain regions are markedly more credulous to misleading advertising (Asp et al., 2012). Difficulties comprehending language or remembering details could also lead individuals to be taken advantage of. Attention lapses can result in victims misunderstanding the scheme they are getting into. These cognitive declines can also be embarrassing and so individuals may go along with a fraudulent scheme to appear competent. These results are in line with what is known about normal age-related cognitive declines in general. Compared to younger adults, older adults experience declines in a wide variety of cognitive domains, including language comprehension, memory, inhibitory control, attention, and cognitive flexibility (Cohen, 1979; Foos and Clark, 2000; Bopp and Verhaeghen, 2005; Jansen, 2006; Thornton and Light, 2006; Slessor et al., 2008; Karbach and Verhaeghen, 2014). Not only does cognitive decline pose a risk factor for fraud victimization, but it is also related to physical and mental health problems and decreased well-being overall (Dolan, 1998; Jansen, 2006). Going beyond these basic mental tasks, declines in fluid intelligence have also been linked to poor capability in making financial decisions which is closely related to fraud (Li et al., 2013; Gamble et al., 2014). Episodic memory, which would be tapped into with items on the MASQ such as “I forget important events which occurred over the last month” has been linked to declines in numeracy (Gamble et al., 2014). Numeracy ability involves comprehending fundamental mathematical operations and has been linked to risky decision making in health (Reyna et al., 2009) and financial domains (Banks et al., 2010). The scale also measures semantic memory, through items like “I find myself calling a familiar object by the wrong name.” Semantic memory declines have been connected to decreases in financial knowledge (Gamble et al., 2014), which in turn has been related to lottery fraud victimization (Consumer Fraud Research Group, 2006). A person who has numeracy difficulties, understanding the nuances of various financial engagements, and well-thought out financial decisions would likely make for an easy fraud target.

#### Social Factors

There were two significant results found for the personality domains when *t*-tests were conducted. First, non-victims scored higher in honesty and humility compared to victims. The reason for the honest and humble individuals to be less likely to become victims of fraud may be their tendency to engage in fair and honest behaviors. Honest individuals have shown to have a heightened sensitivity to fairness (Ashton and Lee, 2008), which in turn may protect them against victimization. When those high in fairness sensitivity are offered a potentially less-than-fair way to earn extra money, they may be less likely to go along with it. Instead, they may be more likely to stick with an existing fair and trusted financial plan. They may be more sensitive to other people acting in devious or unfair ways and avoid these behaviors themselves. In contrast, those who are less honest and humble may not experience as strong of an aversion to unfair financial schemes and may view them as an opportunity to get ahead financially. This tendency to engage in unfair and dishonest behaviors could leave them vulnerable to be taken in by potential scammers.

We also found that victims were less conscientious than non-victims, supporting our original hypothesis. Conscientious individuals tend to be detail-oriented, avoid making impulsive decisions, and are motivated to succeed through hard work (Lee and Ashton, 2004). This kind of person would be more likely to go over a financial proposal carefully, checking for inconsistencies, and taking time to consider potential outcomes. They are driven to achieve through hard work and dedication, and typically are not on the lookout for “get rich, quick” schemes. By contrast, those who are low in conscientiousness are more likely to neglect the small details (Lee and Ashton, 2004), which could cause them to miss tell-tale signs of scams. They also tend to act on impulse. As a result, they may be easily swayed by false promises offered by scammers. These people are also less motivated toward achievement and may find it difficult to maintain discipline in saving for retirement. Thus, taken together, those who are less conscientious become good targets for the sales tactics that fraudsters use to lure their victims.

We also hypothesized that victims would be higher in agreeableness as they may be more likely to comply with fraudster’s fraudulent requests. Our results did not support this hypothesis. This null result may be due to the fact that highly agreeable individuals may be aware of a scammer’s intentions. Thus, these people may not identify themselves as victims because they, though being agreeable, may not be easily deceived. Instead, they may be complying knowingly in order to be cooperative. This possibility needs to be tested with specifically designed studies in the future.

We originally reasoned that highly trustful people would be inclined to believe others generally and therefore put themselves at risk of victimization. Contrary to this original hypothesis, we found no significant relation between trust and fraud victimization. Recent research on trust outside of the fraud literature has suggested that highly trusting people are in fact better at differentiating between trustworthy and untrustworthy others (Carter and Weber, 2010). Given this finding, an alternative possibility is that highly trustful individuals would be less likely to become victims of fraud. This alternative hypothesis is not supported by our results either. Our null findings together with the mixed findings from Anderson (2003) suggest that trust level may be unrelated to victimization. Before accepting this null hypothesis, we must also consider the measure we used to assess trust. Our trust measure asked questions about participants’ trust of groups of people varying along the familiarity dimension from friends, to strangers, and to specific outgroups (i.e., people of different nationality). Future trust measures may consider focusing on participants’ trust of strangers who vary along such dimensions as authority. This is because consumer fraud is generally defined as being committed by people who are strangers to the victim. As fraudsters often impersonate legitimate businesses or act in ways that imply they are authority figures, it would be worthwhile to examine trust toward authority in relation to vulnerability to fraud. For example, future studies could assess victims’ and non-victims’ trust in individual and institutional authorities.

### Combined Contributions to Victimization

In addition to assess each factor’s contribution to fraud victimization individually, we also examined how these factors could contribute to victimization together. When overall cognitive ability, honesty-humility, and conscientiousness were entered into the logistic regression, they were collectively associated with victimization. Further, the overall cognitive ability even uniquely predicted fraud victimization above and beyond the common contribution of all predictor variables. However, when we broke down the overall cognitive ability measure into the three subscales, we did not find any of the three subscale scores to predict fraud victimization above and beyond the common contributions of all predictor variables.

These results together suggest that (1) both social and cognitive factors together play an important role older adults’ likelihood of being victimized by fraud; (2) cognitive ability may have a unique added role in fraud victimization; and (3) the influence of individual specific cognitive factors on fraud victimization is integrative whereby all the three areas of cognition together contribute to fraud victimization. Together, this suggests that one needs to maintain cognitive functioning in all areas throughout the aging process in order to avoid being victimized by fraud. This possibility could also provide some relief for those who are worried about a single aspect of their or their family member’s cognitive functioning. That is, fraud victimization risk due to declines in a single area (e.g., memory) may be mitigated when other aspects of their cognitive abilities are strong or if adaptive strategies are used to maintain an overall high level of cognitive functioning. Future research in fraud victimization should examine the potential compensatory role of each aspect of overall cognitive ability or strategies that older adults use to ensure they are not missing, forgetting, or misunderstanding cues that would alert them to potential scams.

### Limitations and Future Research

This study demonstrated significant relations between cognitive ability, conscientiousness, honesty and humility, and the presence of fraud victimization both in terms of presence/absence. However, the analysis could have benefited from a larger sample size with more representation of males and various ethnicities. A larger sample size could improve the normality distributions on the cognitive variables as well. The analysis was also limited by a procedural error whereby many participants did not provide education information, as well as many missing responses on the language subscale.

Future research should also consider experimental and behavioral methods of examining psychological profiles in relation to fraud. Experimental measures are especially important for examining potential fraud victimization reduction strategies, such as forewarning, which was shown to reduce explicit acceptances of scams (Scheibe et al., 2014). As for behavioral rather than questionnaire methods, this is especially important for measuring cognitive functioning. These should also include other cognitive domains such as processing speed and inhibition. In addition, cognitive measures that take lifelong experience into account should be used. Whereas crystallized intelligence which involves experience and knowledge increases with age, fluid intelligence which involves processes like learning, problem solving, and cognitive flexibility, begins to decline from young adulthood (Salthouse, 2004). Previous research suggests that crystallized intelligence can make up for declines in fluid intelligence when making financial decisions (Li et al., 2013). This interaction between the two types of intelligence should be further examined within the context of fraud victimization. Potential improvements can also be made to the fraud education effort based on lifespan learning theories (Wu et al., 2016) which take into account not only older adults’ biologically based cognitive declines, but also their large knowledge base and well learned behavior patterns.

Self-report measures are often plagued by problems due to the participants’ idiosyncratic interpretations of questions, their unique perception of own behaviors, social desirability bias, and question ordering, just to name a few (Olsen, 1979; de Leeuw et al., 1999; Deevy and Beals, 2013). Behavioral methods would result in more objective data. Furthermore, there is evidence that older adults who experience cognitive declines do not report decreased confidence in making financial decisions, even though they often report lower self-confidence overall in their abilities due to these cognitive declines (Gamble et al., 2014). Subsequent research on this topic should consider using behavioral measures to assess cognitive ability and confidence in financial decision making in people who have been previously victimized by fraud. It would be especially interesting to examine financial confidence in repeat fraud victims.

In addition to the inherent issues with self-report studies, our research is also limited by its cross-sectional design. In the present study, we asked participants to retrospectively report *past* fraud victimization and then used this data to make associations with *current* information in demographic, social, and cognitive domains. While some of these concepts are relatively stable across the lifespan (i.e., a demographic category like race), other measurements may have the potential to change in small and large ways. Because we only looked at fraud after the age of 50 in older adults with no serious cognitive or neurological disorders, we can assume with some confidence that we are still looking at individual differences in cognition and that these global cognitive differences existed before the study took place. Nevertheless, a better research strategy is to use a longitudinal and prospective design, which is unfortunately seldom used in aging research generally (Lichtenberg et al., 2013).

By using the 60-item HEXACO measure, we were unable to examine the individual facets of each personality domain. Of particular interest is the facet of fairness within honesty-humility, in addition to sincerity, greed avoidance, and modesty (Ashton and Lee, 2008). Future research on the topic should use a longer questionnaire to examine fairness within this personality trait or use an independent and comprehensive fairness measure to examine this relation more closely. It would also be interesting to examine conscientiousness more closely by focusing in on its various facets including organization, diligence, perfectionism, and prudence (Lee and Ashton, 2004).

Due to the small sample size and the nature of our participants’ history of victimization, we were unable to compare differences between those who had been victimized by different fraud types. We asked about 15 different types of fraud and had victims in nearly all categories. We know that there are demographic differences between victims of investment and lottery fraud (Consumer Fraud Research Group, 2006) and so there could be psychological differences as well. Researchers conducting prospective studies should consider using a larger sample of victims or target specific types of fraud to compare within-victim differences. A larger sample size may also help to examine differences among those who differ in victimization frequency. In addition, with a large sample size, there is also the opportunity for qualitative and detailed analysis regarding how the fraud occurred. This could provide information on the fraudster’s actions and how the victim dealt with the situation. Non-victims could alternatively provide details about a recent fraud attempt which they successfully rebuffed. This would provide further insight on the behavioral differences between fraud victims and non-victims.

As we learn more about the social and cognitive factors that influence one’s vulnerability to fraud, it is important to consider the interaction of these factors as one makes financial decisions in the face of a potential scam. Being approached by scam artists can be a highly emotional event, as fraudsters try to lure one in with false promises and threats. Future research on fraud victimization should also consider the effects of emotion on older adults’ decision making in such circumstances. In contrast to declines in cognition, emotional processing has shown to be preserved in aging and so may have important implications for how older adults interpret and react to a fraudster’s attempt (Carstensen and Mikels, 2005).

## Conclusion and Implications

The present study showed that lower cognitive ability within the normally aging population and lower levels of honesty and humility and conscientiousness make individuals more vulnerable to being victimized by fraud, with cognitive abilities playing a key role. Cognitive capabilities in old age also play a unique and added role in vulnerability to fraud. Our results supported the previous mixed results in that there were no significant findings in relation to demographics and trust. Importantly, the present findings lay a central foundation for more comprehensive and in depth studies on this theoretically and practically important issue. These findings are highly informative about engagement of preventative measures. Whereas personality is relatively stable throughout our lives, cognitive capabilities have the potential to be trained. Cognitive training and engagement is a popular topic in the aging field as a way to counter-act normal age-related declines which can impair daily performance (Stine-morrow et al., 2014). Potentially, we could use cognitive training techniques to help older adults protect themselves against future victimization. In addition to cognitive decline prevention, aging learning theories can also be incorporated (Wu et al., 2016) to build up a knowledge base to counteract age-related declines. These future studies should significantly advance our understanding of the relation between psychological factors and fraud vulnerability and help develop policies and practices to prevent fraud victimization in older individuals.

## Ethics Statement

This study was carried out in accordance with the recommendations of the Tri-Council Policy Statement: Ethical Conduct for Research Involving Humans (TCPS2), Research Ethics Board at the University of Toronto and the Ryerson Research Ethics Board with written informed consent from all subjects. All subjects gave written informed consent in accordance with the Declaration of Helsinki. The protocol was approved by the Research Ethics Board at the University of Toronto and the Ryerson Research Ethics Board.

## Author Contributions

RJ: designing study, recruitment, data collection, analyzing results, writing final manuscript. SG and LY: recruitment and analysis support, writing final manuscript. KL: designing study, analyzing results, writing final manuscript.

## Conflict of Interest Statement

The authors declare that the research was conducted in the absence of any commercial or financial relationships that could be construed as a potential conflict of interest.
