# Supplementary material for: The Role of Cognition, Personality, and Trust in Fraud Victimization in Older Adults
Source: Front Psychol. 2017 Apr 13;8:588. doi: 10.3389/fpsyg.2017.00588 (PMC5390488; doi:10.3389/fpsyg.2017.00588)
Supplement: Supplementary file 1 [file Data_Sheet_1.docx]

Appendix A

Fraud Questionnaire

Instructions to participants: Please answer the following questions to the best of your knowledge. If you do not feel comfortable answering a question for any reason, just leave it blank and move on to the next question.

Part A.

1. Have you ever been a victim of fraud?
   1. If you have been a victim, please explain briefly (1 sentence).
2. If you have been a victim…
   1. How old (in years) were you when this fraud occurred?
   2. Approximately how much money did you lose due to this scam?
   3. What was the method of contact? (please circle one)

internet/e-mail, telephone; in-person; unknown

Part B.

Instructions to Participants: Please read the description of the hypothetical fraud type then answer the questions following the description. Being *approached* with a certain type of scam means you have been targeted (by receiving an e-mail, by viewing an advertisement, spoken to a door-to-door salesperson, etc.)

- 1. An individual purchased a weight-loss product (i.e. nonprescription drugs, dietary supplements, creams, wraps, etc.) that was promoted as being able to make you lose weight without any dieting or exercise, and the consumer only lost a little of the anticipated weight or lost no weight at all.

1. Have you ever been approached with this type of scam? How many times?
2. If yes… What was the method of contact for this scam? (circle one)

internet/e-mail, telephone; in-person; unknown

1. Have you ever been a victim of this type of scam?
2. If yes…
   1. How old (in years) were you when this occurred?
   2. How much money did you lose in this scam?
   3. What was the method of contact for this scam? (please circle one)

internet/e-mail, telephone; in-person; unknown

1. An individual purchased a medical product that claimed it was a miracle cure for a certain ailment, and the purchaser did not experience most or any of the benefits the product claimed to offer.
2. An individual is asked to pay money (or complied with another request) to receive promised prize or lottery winnings, and did not receive the prize as promised.
3. An individual purchased a work-at home program and did not earn at least one-half of the promised earnings.
4. An individual is asked to donate money or resources to a charity, and later finds out that the charity does not exist or that their donation never reached the promised charity.
5. An individual paid someone who promised to pay off credit card debts for a lesser amount or arrange a lower interest rate on credit card debt and then failed to provide promised services.
6. An individual paid someone for the opportunity to operate their own business and did not receive at least half as much promised or the promised assistance.
7. An individual paid an advance fee to obtain a promised loan or credit card, and never received the credit.
8. An individual received a cheque and sent some of the money back to the sender, and later learned that the cheque was counterfeit.
9. An individual paid someone who claimed to know something either wonderful or terrible in their future. Payment was required for a full report of predictions. As a result of this, the individual lost money.
10. An individual was contacted by a company that appeared to be legitimate and was asked for (and provided) personal details such as bank information or computer passwords.
11. An individual was contacted about an inheritance they could receive if they agreed to pay various legal or banking fees, and they never ended up receiving any inheritance money.
12. An individual signed a contract for a timeshare property that either did not exist or fell well below the promised standards.
13. An individual paid money to someone who falsely claimed to be a friend or family member, told them that they were in an emergency, and asked the individual to send them money.
14. An individual purchased something from a company (online, seen on TV, etc.) and never received the product or received a product that did not measure up to its description.

*Note.* Follow-up questions seen in Part B Question 1 are repeated for each fraud scenario.
